# A New Therapy for Vitiligo Using Fire Needles: A Systematic Review of Evidence from 3618 Subjects

**DOI:** 10.1155/2020/8492097

**Published:** 2020-08-27

**Authors:** Ying Luo, Wei Qian, Ting Dai, Yi Ru, Xiaoying Sun, Le Kuai, Liu Liu, Meng Xing, Qi Zheng, Ying Zhang, Xi Chen, Huaibo Zhao, Bin Li, Xin Li

**Affiliations:** ^1^Department of Dermatology, Yueyang Hospital of Integrated Traditional Chinese and Western Medicine, Shanghai University of Traditional Chinese Medicine, Shanghai 200437, China; ^2^Ningbo Municipal Hospital of TCM, Ningbo, Zhejiang 315012, China; ^3^Institute of Dermatology, Shanghai Academy of Traditional Chinese Medicine, Shanghai 201203, China

## Abstract

**Introduction:**

Fire needle therapy has been reported as an effective treatment for vitiligo. However, current clinical evidence has not been systematically evaluated. The aim of this study was to determine whether fire needle therapy is effective and safe for treating vitiligo.

**Methods:**

Seven databases were searched until October 2019 for randomized controlled trials on fire needle therapy, with and without conventional treatments, versus any type of conventional therapy for treating vitiligo. The RevMan 5.3.5 software was used to perform meta-analysis of the included studies.

**Results:**

Forty-seven trials comprising 3618 patients were included. Fire needle combined with conventional vitiligo treatments had a higher efficacy (risk ratio (RR): 1.55, 95% confidence interval (CI): 1.46–1.65, *P* < 0.00001 and RR: 1.41, 95% CI: 1.24–1.61, *P* < 0.00001, respectively) and a greater effect on restoring the color of the area of the skin lesion (mean difference (MD): 3.40, 95% CI: 2.11–4.69, *P* < 0.00001), increasing the pigment point of vitiligo (MD: 0.83, 95% CI: 0.54–1.13, *P* < 0.00001) and improving the cytokine level (MD: 8.10, 95% CI: 6.94–9.27, *P* < 0.00001) and effectual time (MD: −4.76, 95% CI: −7.33 to −2.19, *P*=0.0003) than traditional methods. Limb lesions (RR: 1.60, 95% CI: 1.31–1.95, *P* < 0.00001) were more effectively treated when the treatments included fire needles, whereas the therapeutic effect of fire needles on either the head and neck (RR: 1.13, 95% CI: 0.78–1.64, *P*=0.52) or torso lesions (RR: 1.22, 95% CI: 0.82–1.81, *P*=0.33) was not significantly different compared to that without fire needles. No statistically significant differences in adverse effects (RR: 1.15, 95% CI: 0.89–1.49, *P*=0.28) and recurrence rates (RR: 0.90, 95% CI: 0.17–4.92, *P*=0.91) during the follow-up period were observed between treatment with and without fire needles.

**Conclusions:**

Fire needle therapy combined with other conventional treatments is useful in treating vitiligo. Further studies with larger sample sizes should be performed to make a conclusive judgment. This trial is registered with CRD42018094918.

## 1. Introduction

Vitiligo is a frequently acquired depigmentation disease of localized or generalized skin and mucosa. A typical pathological feature of the disease is a lack of melanocytes and melanin granules, and it is characterized by whitening of lesions on the skin [1]. Approximately 1% to 2% of people globally have vitiligo [2], including both adults and children. The cause of vitiligo is still unclear, but it is considered to be related to heredity, immunity, mental and neurological causes, melanin self-destruction, and oxidative stress, among other causes [3]. Vitiligo could lead to serious loss of normal appearance, which may result in negative effects on learning, work, social interaction, and the marriage status of patients. Conventional treatments for vitiligo include topical corticosteroids, calcitonin inhibitors, phototherapy, and transplantation [4]. These treatments have severe side effects including chemical drug toxicity and phototherapy diffusion in varying degrees [5]. Consequently, there is an urgent need for new approaches to treatment.

Vitiligo is called “Baidian” or “Baibofeng” in traditional Chinese medicine (TCM), which has a long history of treatment for the disease. Fire needle therapy, recorded as early as the time of the Yellow Emperor (475–221 BC), has been an integral part of TCM and the acupuncture method. In the procedure, a special nontoxic stainless-steel needle is inserted into the acupoint after heating (Supplementary file 1), thus improving localized blood flow in the skin lesions and promoting the formation of melanin [6], which has been proved to be effective for treating vitiligo [7, 8]. However, to our knowledge, no systematic review has evaluated the benefits of fire needle therapy in patients with vitiligo. Therefore, we conducted this systematic review to comprehensively assess the clinical efficacy and safety of this classic treatment for vitiligo, in order to develop and supplement the therapeutic approaches available.

## 2. Methods

This systematic review was performed following the Cochrane Handbook for Systematic Reviews of Interventions [9], presented under the Preferred Reporting Items for Systematic Reviews and Meta-analyses (PRISMA) guidelines (Supplementary file 2).

### 2.1. Inclusion Criteria

In this analysis, we included randomized controlled trials (RCTs) that used interventions of either fire needles alone or fire needles combined with conventional therapy, compared with any conventional therapy for vitiligo without fire needles. We included studies in which the pricking area was on the Ashi points (local skin lesions). The patients must have been diagnosed with vitiligo, regardless of sex, age, and ethnicity.

### 2.2. Outcomes

The primary outcome measured in this study was the total effectiveness rate for the duration of treatment, defined as the rate of restoration of the vitiligo lesions to normal color. This was divided into four categories: (i) cured: vitiligo had subsided with the skin color restored to normal; (ii) markedly effective: vitiligo had partially subsided or reduced, with more than 50% of the skin area restored to the normal skin color; (iii) effective: part of the vitiligo had subsided or reduced; and (iv) ineffective: vitiligo had been pigmented, regenerated, or expanded with reduction of the healing number.

If the trials used the number of patients as the unit for statistical outcomes, the effectiveness rates were calculated using the following *NP formula*: effectiveness rate = (number of *cured* patients + number of *markedly effective* patients)/number of total patients × 100%. If the trials used the number of vitiligo skin lesions as the unit for statistical outcomes, the effectiveness rates were calculated by the following *NVSL formula*: effectiveness rate = (number of *cured* vitiligo skin lesions + number of *markedly effective* vitiligo skin lesions)/number of total vitiligo skin lesions × 100%.

The secondary outcomes included total restoration of the area's color, the total increased pigment point, changes in the cytokine level, effectual time, rate of the therapy's effectiveness in different locations, and adverse effects, as well as recurrence rates. The pigment point was defined as follows: 0 *points,* pure white skin lesions without any pigment production; 1 *point,* pale white skin with a little pigmentation; 2 *points,* pale brown skin lesions with a lot of pigmentation; and 3 *points,* skin damage with tan, basic, or near normal skin color.

### 2.3. Selection of Studies and Data Extraction

We searched the Cochrane Library, Excerpta Medica data BASE (EMBASE), PubMed, the China National Knowledge Infrastructure Database, Chinese Scientific Journals Full Text Database, Wanfang Data Knowledge Service Platform, and the Chinese Biomedical Literature Service System. We included papers dating from the earliest citation in the databases until October 2019. The search terms included “vitiligo” or “leukoderma,” combined with “fire needle”.

Two investigators (R.Y. and Z.Y.) independently screened the studies according to the inclusion criteria and extracted the information based on self-designed data-extraction templates, which included the first author, study characteristics (i.e., year, duration, setting, and design), participant characteristics (i.e., mean age, sample size, TCM syndrome, and interventions), and measured outcomes.

### 2.4. Risk of Bias Assessments

The Cochrane Handbook [9] was used to evaluate the methodological quality of each included study in terms of the following characteristics: random sequence generation, allocation concealment, blinding of participants and personnel, blinding of the outcome assessment, incomplete outcome data, selective reporting, and other biases. The terms “low,” “unclear,” and “high,” respectively, referred to low, uncertain, and high risks of bias. The results were cross checked by two investigators (Z.Q. and L.L.), and the disagreements were settled by discussion between them.

### 2.5. Data Analyses

The RevMan 5.3.5 software (Cochrane Community, London, United Kingdom) was used for the data analyses. Dichotomous data are expressed as risk ratios (RRs) with 95% confidence intervals (CIs). Continuous data are expressed as mean differences (MDs) with 95% CIs. For each meta-analysis, we evaluated the statistical heterogeneity. If the trial had acceptable homogeneity (*P* > 0.1, *I*^2^ < 50%), a fixed-effects model was applied; if there was statistical heterogeneity (*P* < 0.1, *I*^2^ > 50%), we used a random-effect model. Clinical heterogeneity was assessed by reviewing the differences in the distribution of participants' characteristics between trials (i.e., age, sex, and disease duration).

## 3. Results

### 3.1. Description of Studies

**Figure 1:**
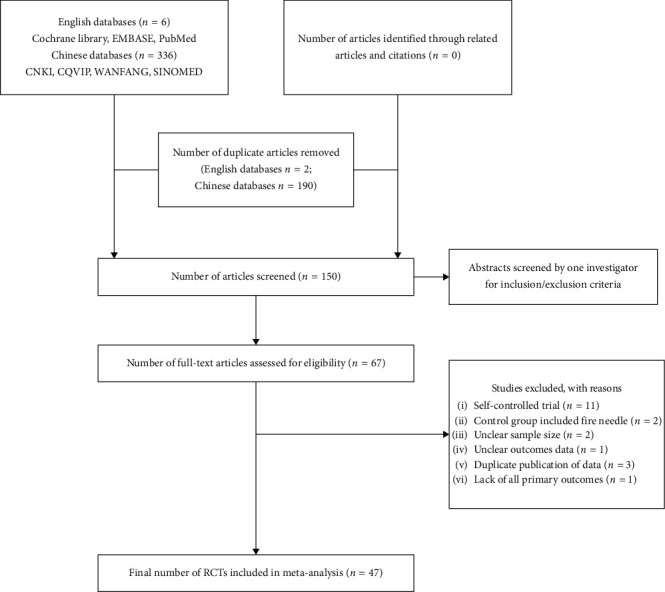
Flowchart of study selection. RCT, randomized controlled trial; EMBASE, Excerpta Medica dataBASE.

After performing primary searches of the seven databases, we identified 342 potential articles. We excluded 293 as they did not conform to the inclusion criteria. Eventually, 47 trials with a total of 3618 patients [10–56] were included in this review (Figure 1). Among the included articles, eight [29–32, 46, 47, 50, 55] were unpublished master's theses.

The characteristics of the included trials are presented in Table 1. All 47 trials [10–56] were performed in China. Only one trial [31] was a multicenter study, and the rest were single-center studies. The study population included children and adults. All included patients had a confirmed diagnosis of vitiligo. In the diagnosis of TCM syndromes, eight trials [12, 19, 21, 36, 46, 48, 52, 53] only included patients with “Qi-stagnancy and blood stasis,” “liver-kidney yin deficiency,” “blockage of the vessel,” or “liver depression and Qi-stagnancy” TCM patterns, as described in the TCM dialectic. Interventions included the combination of fire needle therapy on the Ashi points (local skin lesions) and conventional treatments (308-nm excimer laser, narrow-band ultraviolet B light, Western medicine ointment, and other TCM methods). The control participants used conventional treatments, without the use of fire needles. Courses of treatment ranged from 2 to 6 months.

Twelve trials [29–32, 35, 36, 42, 50, 51, 54–56] reported data on the area around the skin lesions before and after the treatments. Six trials [32, 37, 52, 54–56] reported data on the total pigment point of the skin lesions before and after the treatments. Five trials [35, 37, 38, 52, 56] reported various changes of the cytokine level. Three trials [26, 36, 45] reported the effectual time of treatments in both the experimental and control groups. Four trials [11, 21, 23, 26] examined the effect of the treatment on skin lesions placed at different locations in both the experimental and control groups. Eight trials [14, 28, 31, 32, 39, 52, 54, 55] reported follow-up data, and 41 trials [10, 11, 13–24, 26–37, 39–43, 46–53, 55, 56] reported adverse events.

### 3.2. Methodological Quality

**Figure 2:**
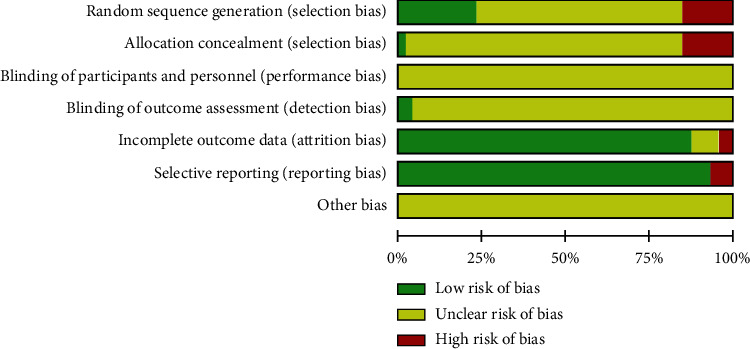
Risk of bias graph.

The Cochrane risk of bias for all 47 trials is shown in Figure 2. Two trials [31, 32] reported using a sample-size calculation method. Eleven trials [31–34, 37, 38, 43, 47, 49, 54, 55] used randomization procedures that included random-number tables or the computer randomization method, but only one [32] reported the concealing allocation adequately. Since the interventions involved fire needles, blinding could not be applied to the patients and researchers in all 47 trials; thus, only two trials [31, 32] used blinding to assess the outcomes. Four trials [10, 13, 19, 20] did not report the loss of patients to follow-up, while only one trial [22] used an intention-to-treat analysis.

### 3.3. Meta-Analysis of Primary Outcomes

#### 3.3.1. Total Effectiveness Rate

Forty-four trials [10–12, 14, 16–46, 48–56] used the number of patients as the unit of statistical outcomes with the total effectiveness rate counted using the *NP formula*. Of the 44 trials, nine [12, 14, 17, 20, 23, 24, 39, 45, 49] compared the use of fire needles and a 308-nm excimer laser with a 308-nm excimer laser alone; eight trials [10, 16, 18, 21, 35, 36, 41, 48] compared the use of fire needles and other TCM methods with other TCM methods alone; three trials [28, 31, 54] compared the use of fire needles and tacrolimus ointment with tacrolimus ointment alone; 14 trials [11, 19, 22, 27, 33, 37, 38, 40, 42, 44, 46, 53, 55, 56] compared the use of fire needles and other treatments with other treatments alone; three [25, 26, 50] compared the use of fire needles and other TCM methods with both the phototherapy and the same TCM methods; and seven trials [29, 30, 32, 34, 43, 51, 52] compared the use of fire needles and other TCM methods with other traditional treatments.

**Figure 3:**
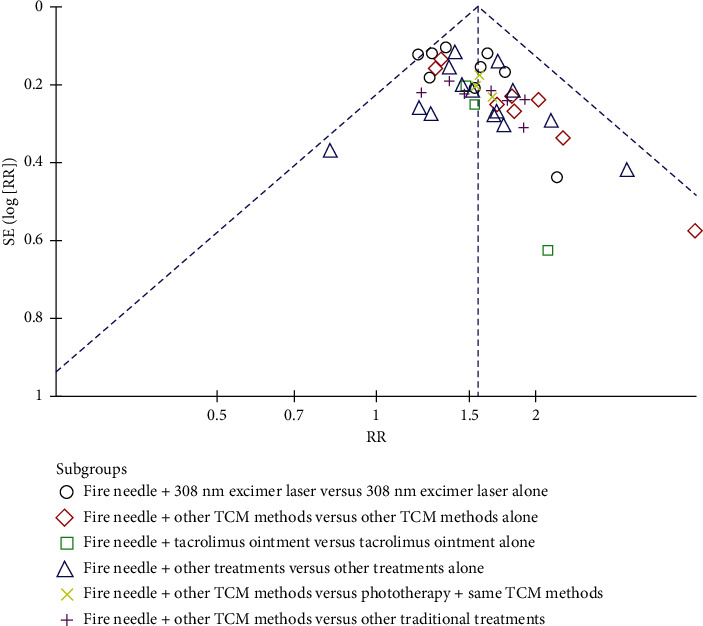
Funnel plot of the total effectiveness rate according to the NP formula. TCM, traditional Chinese medicine; NP formula, effectiveness rate = (the number of cured patients + the number of markedly effective patients)/the number of total patients × 100%.

The subgroup analysis with a fixed-effects model is shown in Table 2(A and B). There was a superior difference in the effect of fire needle therapy with a 308-nm excimer laser *vs.* that of the 308-nm excimer laser alone (RR: 1.46, 95% CI: 1.32–1.61, *P* < 0.00001); fire needle therapy and other TCM methods *vs*. other TCM methods alone (RR: 1.66, 95% CI: 1.43–1.94, *P* < 0.00001); fire needle therapy and tacrolimus ointment *vs.* tacrolimus ointment alone (RR: 1.57, 95% CI: 1.15–2.14, *P*=0.005); fire needle therapy and other treatments *vs.* other treatments alone (RR: 1.56, 95% CI: 1.39–1.74, *P* < 0.00001); fire needle therapy and other TCM methods *vs.* both the phototherapy and the same TCM methods (RR: 1.58, 95% CI: 1.25–2.00, *P*=0.0001); and fire needle therapy and other TCM methods *vs*. other traditional treatments (RR: 1.59, 95% CI: 1.34–1.88, *P* < 0.00001). We analyzed the publication bias of the aforementioned trials (Figure 3) and found that publication bias existed but was small.

The remaining three trials [13, 15, 47] used the number of vitiligo skin lesions as the unit of statistical outcomes with the total effectiveness rate counted using the *NVSL formula*. All three trials involved interventions of fire needle therapy and other treatments *vs.* other treatments alone (specific types in Table 1 (A), (B)). Using a fixed-effects model (Table 2), we found that there was an obvious difference in the effect of the therapies (RR: 1.41, 95% CI: 1.24–1.61, *P* < 0.00001).

### 3.4. Meta-Analysis of the Secondary Outcomes

#### 3.4.1. Total Restoration of the Area's Color

Twelve trials [29–32, 35, 36, 42, 50, 51, 54–56] reported data on the total area of the skin lesions both before and after treatment. In a comparison of the restored color on skin lesions using random-effects modeling (Supplementary file 3), we found that treatment including fire needles had a significantly greater effect in restoring the color of the skin than traditional methods without fire needles (MD: 3.40, 95% CI: 2.11–4.69, *P* < 0.00001).

#### 3.4.2. Total Increased Pigment Point

Six trials [32, 37, 52, 54–56] reported data on the total pigment point of the skin lesions both before and after treatment. In a comparison of the increased pigment point of these skin lesions using random-effects modeling (Supplementary file 4), we found that treatment including fire needles had a significantly greater effect in increasing the pigment point of the skin than traditional methods without fire needles (MD: 0.83, 95% CI: 0.54–1.13, *P* < 0.00001).

#### 3.4.3. Changes in the Cytokine Level

Five trials [35, 37, 38, 52, 56] reported various changes in the cytokine level, which demonstrated that fire needle therapy combined with traditional treatments could affect vitiligo-related cytokines. Two trials [38, 56] reported the serum interleukin- (IL-) 17 level both before and after treatment. In a comparison of changes in the serum IL-17 level using a fixed-effects model (Supplementary file 5), we found that treatment including fire needles had a significantly greater effect on reducing the serum IL-17 level than traditional methods without fire needles (MD: 8.10, 95% CI: 6.94–9.27, *P* < 0.00001).

#### 3.4.4. Effectual Time

Three trials [26, 36, 45] reported the effectual time of treatments in both experimental and control groups. In a comparison of the effectual times using random-effects modeling (Supplementary file 6), we found that treatment including fire needles had a significantly therapeutic effect in a shorter time than traditional methods without fire needles (MD: −4.76, 95% CI: −7.33 to −2.19, *P*=0.0003).

#### 3.4.5. Rate of the Therapy's Effectiveness in Different Lesion Locations

Three trials [21, 23, 26] examined the effectiveness of treating lesions with different locations in both the experimental and control groups using the *NVSL formula*. With random-effects modeling, we discovered that interventions including fire needles for lesions placed at limbs had a better effect than interventions that did not use fire needles (RR: 1.60, 95% CI: 1.31–1.95, *P* < 0.00001) (Supplementary file 7). At the same time, when the conventional treatments with and without fire needles on either head and neck lesions (RR: 1.13, 95% CI: 0.78–1.64, *P*=0.52) or torso lesions (RR: 1.22; 95% CI: 0.82–1.8, *P*=0.33) were compared, there was no statistically significant difference.

Only one trial [11] examined the effectiveness of treating lesions placed at different locations in both the experimental and control groups using the NP formula, which concluded that treatments (“Bailing pian” of Chinese patent drug and NB-UVB) with fire needles on the head, neck, torso, and limbs had a better effect than without fire needles.

#### 3.4.6. Adverse Effects

**Figure 4:**
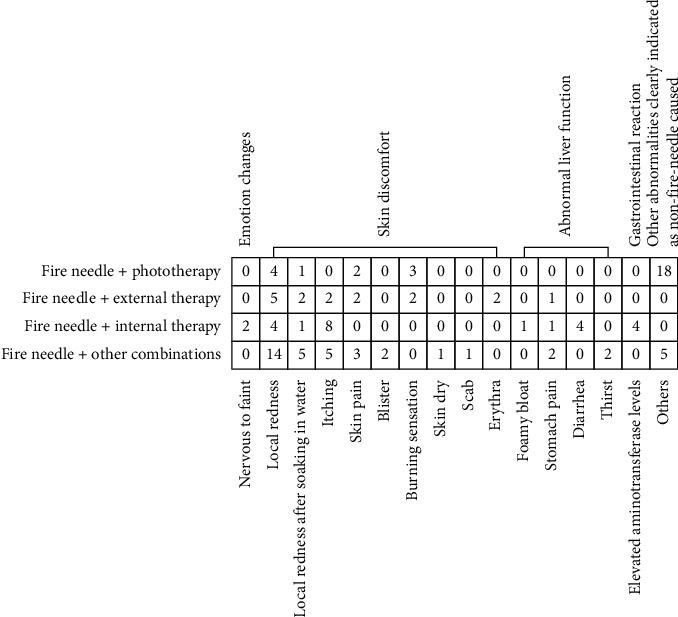
Statistics of adverse effects in each experimental group.

Forty-one trials [10, 11, 13–24, 26–37, 39–43, 46–53, 55, 56] mentioned adverse effects; eight trials [16–18, 28, 40, 49, 52, 56] reported that there was no adverse effect in either the experimental or control groups, and one trial [46] reported adverse effects but did not mention in which groups they occurred. We analyzed the remaining 32 trials with a fixed-effects model (Supplementary file 8) and found that there was no statistically significant difference between the treatments with and without fire needles (RR: 1.15, 95% CI: 0.89–1.49, *P*=0.28). No serious adverse effects were reported in any of the trials. Mild adverse effects, such as a burning sensation, local redness, and itching of the skin, were tolerable, and they tended to disappear without treatment or after symptomatic treatment (Figure 4).

#### 3.4.7. Recurrence Rates

Eight trials [14, 28, 31, 32, 39, 52, 54, 55] mentioned the recurrence rate during the follow-up period. Two trials [14, 31, 39, 52, 55] reported that no patients experienced any relapse. With a fixed-effects model, we found that two trials [28, 32, 54] (Supplementary file 9) showed there was no difference in the recurrence rate between treatments with and without fire needles (RR: 0.90, 95% CI: 0.17–4.92, *P*=0.91).

## 4. Discussion

The data in this study indicated that when compared with traditional methods, fire needles combined with conventional treatments for vitiligo had a higher efficiency, as well as a shorter time before taking effect, and a greater ability to reduce serum cytokines related to vitiligo, restore the color, and increase the pigment point of skin lesions. Limb lesions were more effectively treated when the treatments included fire needles, whereas the effect of treatment on lesions placed at the head, neck, and torso was not significantly different between treatments that used and those that did not use fire needles. There was no statistically significant difference in adverse effects and the recurrence rate during the follow-up period between the methods that included and those that did not include fire needles.

Vitiligo, a disease with a high incidence, is difficult to treat. The main symptoms of skin leukoplakia affect patients' work, daily life, and mental state, thereby leading to serious problems with sleeping and learning. Improvement in the therapeutic outcomes of the condition would improve patients' quality of life [57]. We based this study on the TCM theory, verifying that fire needle therapy, which had some therapeutic effect without serious side effects and could be applied directly to the skin, might be a new way to treat vitiligo.

Chinese medicine proposes that when the human body is greatly stimulated by spirit or material, the “peak potential” of the body's electrical activity is increased, and the electrical activity of the skin damaged by tyrosinase is decreased. The binding of tyrosinase oxidative dopamine with protein is affected, thus destroying the formation of melanosomes. With abnormal neural electrical activity, the function of the cutaneous nerve crest is damaged, along with affected melanocyte formation and metabolism, thereby causing vitiligo [58]. In recent years, fire needle therapy has been proved to be an effective and almost side-effect-free external treatment in TCM [59, 60], while clinical research on the application of fire needle therapy for vitiligo has become a popular treatment method. A previous study confirmed that, after the treatment of vitiligo with fire needles, color on the lesions emerged; in the meantime, using a confocal laser scanning microscope, it was observed that dendritic melanocytes appeared in the skin lesions, the pigment content gradually increased and, then, formed a complete pigment ring, and the melanin ring gradually became bright [57]. Laboratory studies also demonstrated that fire needle therapy could improve the recoloration rate of hair follicles and promote the recoloration in refractory locations of acro-limbs. It has been reported that fire needle therapy could help to improve localized blood flow, expand the capillaries, and accelerate the circulation of blood. Nutrition could be strengthened to stimulate the activity of tyrosinase, thus facilitating the formation of melanin [6]. However, acupuncture needling into the skin produces localized inflammatory stimulation that might activate amelanotic melanocytes (AMMC) in the hair root sheath outside the hair follicle, thereby promoting the differentiation of AMMCs to normal melanocytes [61]. The efficacy of fire needle therapy for vitiligo involves the immunomodulatory mechanism that is associated with regulating the proportion of Th17 cells, IL-17, and IL-23 cytokine levels, as well as the function of T lymphocytes [62, 63].

This study had some limitations that should be acknowledged. The studies included in this review generally had poor methodologies, which could have caused bias. Not all studies mentioned the method of random order generation. In addition, only two trials [31, 32] mentioned the blinding method and one trial [32] utilized concealment of allocation. Moreover, the difficulties associated with blinding in studies of acupuncture treatment led to a low methodological quality, causing possible selection bias in RCTs. The safety and efficacy of acupuncture treatment is attracting increasing attention from the domestic and international medical communities. Nevertheless, acupuncture, particularly fire needle therapy, differed significantly in characteristics from drug treatment; thus, effective blinding and the selection of an appropriate placebo had long been recognized as extremely challenging [64]. It was quite clear that the development of more appropriate RCT designs and protocols was an urgent problem that acupuncture therapy faced. Since fire needle therapy was a TCM treatment, all of the studies included were performed in China. The therapy's application in other countries and regions must be studied further. Few studies mentioned the recurrence of lesions and the efficacy of the treatment for lesions placed at different locations, which might have brought about a difference in the results. Additionally, most existing studies had small sample sizes, which might have produced a high level of bias.

The results of this review showed that there is still a lack of well-designed studies on fire needle therapy in the treatment of vitiligo. Since severe adverse effects were not reported in the included studies and the curative effect of therapy with fire needles was definite, we believe that it would be worthwhile to conduct further rigorously designed RCTs with larger sample sizes and high methodological quality on fire needle therapy in the treatment for vitiligo in the future.

## 5. Conclusions

Fire needle therapy combined with other conventional treatments had some effect on vitiligo lesions, were not associated with any side-effects, and could be administered in a straightforward manner. More high-quality studies with larger sample sizes should be conducted to make a conclusive judgment of its broader application for treatment.

## Figures and Tables

**Table 1:** Characteristics of the included trials.

The First author, year of publication	Sample size		Sample age (mean [SD] year)				Disease duration (mean [SD])				TCM syndrome	Were baseline data comparable?	Intervention		Outcomes	Duration of treatment	Follow-up (months)
	E	C	E		C		E		C				E	C			
Bo Z. F, 2017 [10]	23	23	32.54 [11.61]		32.75 [10.26]		5.28 [2.35] years		5.53 [2.42] years		Qi-stagnancy and blood stasis	Yes	(A) (1) Fire needles (once/7 days); (2) plus (B)	(B) “Shu Gan Huo Xue” decoction of TCM (0.5 dose/once, twice/1 day)	TER, ADs	12 weeks	NR
Chen G. F, 2017 [11]	27	26	29.5 [1.3]		31.5 [1.5]		NR		NR		NR	Yes	(A) (1) Fire needles (once/14 days); (2) plus (B)	(B) (1) “Bailing pian ” of Chinese patent drug (0.33 g^*∗*^4/once, 3 times/1 day); (2) NB-UVB (twice/7 days, Shanghai SIGMA Co., SS-03B)	TER, DPER, ADs	6 months	NR
Kuang W. B, 2013 [12]	96	90	8.7		9.2		NR		NR		NR	Yes	(2) Plus (B)	(B) 308-nm excimer laser (twice/7 days, Shenzhen GSD Co.)	TER	12 weeks	NR
Luo G. P, 2016 [13]	72	75	23.38 [7.56]		27.85 [12.64] months		NR		NR		NR	Yes	(A) (1) Fire needles (once/14 days);	(B) Halometasone cream (twice/1 day, Hongkong Bright Future Co., HC20100039)	TER, DPER (E), DAER (E), DDER (E), DCER (E), ADs	6 months	NR
Ren L. S, 2016 [14]	50	50	26.5		29.5		3.0 years		2.5 years		NR	Yes	(A) (1) Fire needles (once/7 days); (2) plus (B)	(B) 308-nm excimer laser (twice/7 days, Italy DEKA Co., MEL308-nm)	TER, number of treatments, ADs, FUP	12 weeks	3
Xie H. L, 2017 [15]	38	34	25.46 [15.52]		26.85 [8.47]		29.43 [14.26] months		28.69 [15.42] months		NR	Yes	(A) (1) Fire needles (once/7 days); (2) plus (B)	(B) 0.1% tacrolimus ointment (twice/1 day, Astellas (China) Co.)	TER, DPER (E), DAER (E), DDER (E), ADs	12 weeks	NR
Xu D. P, 2016 [16]	29	29	31.3		33.6		NR		NR		Liver-kidney yin deficiency; blockage of the vessel	Yes	(A) (1) Fire needles (once/7 ～ 10 days); (2) plus (B)	(B) “Huo Xue Bu Shen Xiao Bai” decoction of TCM (0.5 dose/once, twice/1 day)	TER, ADs	6 months	NR
Yu C. D, 2017 [17]	30	28	31.5		30.6		3.0 years		2.5 years		NR	NR	(A) (1) Fire needles (once/7 days); (2) plus (B)	(B) 308-nm excimer laser (twice/7 days, USHIO (Japan) Co., TheraBeam UV308)	TER, ADs	12 weeks	NR
Zhao Y, 2015 [18]	39	39	32.1		34.6		NR		NR		Qi-stagnancy and blood stasis	Yes	(A) (1) Fire needles (once/7 days); (2) plus (B)	(B) “Qu Bai Yi HAO” decoction of Chinese drug preparation (200 ml/once or twice/1 day)	TER, ADs	16 weeks	NR
Zhou R. X, 2012 [19]	100	100	35.6		34.5		5.5 years		5.1 years		NR	Yes	(A) (1) Fire needles (separate from (B), once/2 days); (2) plus (B)	(B) NB-UVB (once/2 days, German Waldmann Co., UV100 L)	TER, ADs	120 days	NR
Zhu Y. Y, 2016 [20]	43	40	22		24		2.7 years		3.4 years		NR	Yes	(A) (1) Fire needles (once/7 days); (2) plus (B)	(B) 308-nm excimer laser (twice/7 days, 308-nm XeCl from America)	TER, ADs	12 weeks	NR
Yang D. K, 2014 [21]	30	29	35.5		32.5		7.3 years		7.2 years		NR	Yes	(A) (1) Fire needles (once/5–7 days); (2) plus (B)	(B) “Fu Fang Zi Gui Pian” of Chinese patent drug (1.6 g/once, 3 times/1 day)	TER, DPER, ADs	2 months	NR
Mai L. X, 2017 [22]	31	23	34.81 [8.85]		34.04 [11.17]		33.42 [38.84] months		34.30 [52.52] months		NR	Yes	(A) (1) Fire needles (once/14 days); (2) plus (B)	(B) (1) NB-UVB (twice/7 days, TL20 W/01 lamp tube, wave length 311-313-nm, wave peak 311-nm); (2) Vitamin B complex tablets (2#/once, 3 times/1 day)	TER, ADs	16 weeks	NR
Dun G, 2016 [23]	50	50	33.5				48.6 months				NR	Yes	(A) (1) Fire needles (once/7 days); (2) plus (B)	(B) 308-nm excimer laser (twice/7 days, Italy DEKA Co., MEL308-nm)	TER, DPER, ADs	6 months	6
Jiang M. J, 2016 [24]	30	30	29.5			30.2	2.6 years			2.5 years	NR	Yes	(A) (1) Fire needles (once/7 days); (2) plus (B)	(B) 308-nm excimer laser (twice/7 days, Wuhan MIRACLE LASER Co., MEL308-nm)	TER, ADs	12 weeks	NR
Jing L. H, 2015 [25]	31	29	28.7			29.2	2.3 years			2.7 years	NR	Yes	(A) (1) Fire needles (once/7 days); (2) “Bai Dian Yin” decoction of Chinese herbal granules (twice/1 day)	(B) (1) NB-UVB (twice/7 days, Shanghai SIGMA Co., SS-05B-40); (2) “Bai Dian Yin” decoction of Chinese herbal granules (twice/1 day)	TER	12 weeks	NR
Li X. S, 2017 [26]	60	60	25.6 [11.21]			25.3 [10.63]	2.6 [1.7] years			2.8 [1.2] years	NR	Yes	(A) (1) Fire needles (determined for every patient); (2) “Zi Tong Xiao Bai” of Chinese patent drug (adult: 6#/once, 3 times/1 day; children: 3#/once, 3 times/1 day)	(B) (1) NB-UVB (twice/7 days); (2) “Zi Tong Xiao Bai” of Chinese patent drug (adult: 6#/once, 3 times/once/1 day; children: 3#/once, 3 times/1 day)	TER, DPER, ET, ADs	3 months	NR
Wang W. L, 2017 [27]	39	39	32.50 [8.30]			33.79 [9.12]	28.16 [16.05] months			27.28 [20.21] months	NR	Yes	(A) (1) Fire needles (once/7 days); (2) plus (B)	(B) (1) NB-UVB (once/3 days, German Waldmann 311); (2) 0.1% tacrolimus ointment (twice/1 day, Astellas (China) Co.)	TER, ADs	4 months	NR
Yang D, 2017 [28]	31	31	32.5 [16.5]			30.52 [15.55]	NR			NR	NR	Yes	(A) (1) Fire needles (once/14 days); (2) plus (B)	(B) 0.1% tacrolimus ointment (twice/1 day)	TER, ADs, FUP	12 weeks	2
Liu J. J, 2015 [29]	31	29	32.26 [8.450]			32.72 [8,071]	36.13 [23.082] months			33.90 [18.074] months	NR	Yes	(A) (1) Fire needles (once/7 days); (2) “Ru Yi Hei Bai” of Chinese medicinal powder (6 g/once, 3 times/1 day)	(B) (1) “Bai Bo” of Chinese medicinal pill (5 g/once or twice/1 day); (2) “Ka Li Zi Ran Ding” of Chinese herbal tincture (3-4/1 day); (3) UVA once or twice/1 day, Xinjiang WEIATANG)	TER, DPER (ungrouped), TSLA, DPSLA, ADs	2 months	NR
Wang X, 2016 [30]	36	36	33.66 [8.31]			33.73 [8.57]	22.59 [9.80] months			22.65 [9.31] months	NR	Yes	(A) (1) Fire needles (once/7 days); (2) “Tong Luo Qu Bai” dedoction of TCM (0.5 dose/once or twice/1 day)	(B) (1) “Bai Dian Feng ” of Chinese medicinal pill (12 g/once or twice/1 day); (2) “Ka Li Zi Ran Ding” of Chinese herbal tincture (3-4 times/1 day); (3) UVA (once or twice/1 day, Xinjiang WEIATANG)	TER, TSLA, HAMD, ADs	6 months	NR
Yang M, 2017 [31]	18	19	37.11 [11.97]			38.47 [9.92]	8.92 [10.10] years			8.41 [7.62] years	NR	Yes	(A) (1) Fire needles (1^st^–4^th^ weeks: once/3 days; 5^th^–8^th^ week: once/5 days; 9^th^–12^th^ weeks: once/7 days); (2) plus (B)	(B) 0.1% tacrolimus ointment (twice/1 day, Astellas (China) Co., J20100015)	TER, TSLA, S-CLSM, FUP, ADs	12 weeks	1
Zhang J. Y, 2016 [32]	35	35	32.53 [10.920]			32.37 [9.103]	2.06 [1.42] years			2.05 [1.21] years	NR	Yes	(A) (1) Fire needles (once/7 days); (2) moxa-moxibustion (15 min/once, once/1 day, location: local skin lesion, Xiabai acupoint, Dianfeng acupoint, Zusanli acupoint)	(B) Halometasone cream (once/1 day; intermittent treatment: stopping for 1 week after using for 1 week, German Waldmann Co.)	TER, TRCA, TSLA, TPP, FUP, LA, ADs, CA	3 months	3
Du Y. S, 2019 [33]	40	38	45.49 [3.08]			45.56 [3.12]	1.82 [0.57] years			1.86 [0.54] years	NR	Yes	(A) (1) Fire needles (once/7 days); (2) cotton moxibustion (once/7 days); (3) plus (B)	(B) NB-UVB (three times/7 days, German Waldmann Co.)	TER, ADs	16 weeks	NR
He G, 2018 [34]	28	28	26 [0.3]			27 [0.2]	2.8 [0.6] years			2.6 [0.3] years	NR	Yes	(A) (1) Fire needles (once/7 days); (2) moxa-moxibustion (30 min/once, once/1 day, location: local skin lesion)	(B) Tacrolimus ointment (>10 years old patients: 0.1%; ≤10 years patients: 0.03%; twice/1 day, Astellas (China) Co.)	TER, ADs	12 weeks	NR
Lin M, 2019 [35]	48	47	34.36 [5.44]			33.94 [5.23]	5.45 [2.43] years			5.64 [2.38] years	NR	Yes	(A) (1) Fire needles (once/7 days); (2) plus (B)	(B) (1) “Bu Shen Huo Xue” decoction of TCM (0.5 dose/once, twice/1 day); (2) tacrolimus ointment (twice/1 day)	TER, TSLA, CL, ADs	12 weeks	NR
Liu Z. H, 2019 [36]	60	60	31.32 [6.11]			31.33 [6.45]	10.21 [7.13] years			10.29 [7.1] years	Liver-kidney yin deficiency; Qi-stagnancy and blood stasis; Liver depression and Qi-stagnancy	Yes	(A) (1) Fire needles (once/7 days); (2) plus (B)	(B) “Ka Li Zi Ran Ding” of Chinese herbal tincture, then sunbathing for 30 min (3-4 times/1 day)	TER, TSLA, ET, ADs	NR	NR
Tian L. Y, 2018 [37]	40	40	38.4 [6.9]			38.7 [6.3]	5.1 [1.4] years			5.5 [1.2] years	NR	Yes	(A) (1) Fire needles (once/14 days); (2) plus (B)	(B) NB-UVB (twice/7 days, Shanghai SIGMA Co., SS-01)	TER, CL, TPP, ADs	2 months	NR
Wang W. L, 2018 [38]	50	50	36.03 [7.05]			35.37 [6.59]	NR			NR	NR	Yes	(A) (1) Fire needles (once/7 days); (2) plus (B)	(B) (1) NB-UVB (three times/7 days, German Waldmann Co.); (2) “Bai Dian Feng” decoction of Chinese herbal granules (three times/1 day)	TER, CL	12 weeks	NR
Xu C. Q, 2018 [39]	40	40	20.83 [13.47]			20.19 [12.28]	28.67 [12.34] months			29.43 [14.26] months	NR	Yes	(A) (1) Fire needles (once/7 days); (2) plus (B)	(B) 308-nm excimer laser (Shenzhen GSD Co.)	TER, FUP, ADs	12 weeks	3 months
Yin Y. Q, 2018 [40]	32	31	25.16 [3.58]			25.30 [3.61]	2.92 [1.08] years			3.01 [1.05] years	NR	Yes	(A) (1) Fire needles (once/14 days); (2) plus (B)	(B) Halometasone cream (twice/1 day, Hongkong Bright Future Co., HC20150050)	TER, DPER	6 months	NR
Zhao X, 2018 [41]	49	49	31.5 [3.1]			30.8 [2.9]	5.2 [1.3] years			5.5 [1.4] years	NR	Yes	(A) (1) Fire needles (once/10 days); (2) plus (B)	(B) “Bu Shen Huo Xue” decoction of TCM (0.5 dose/once, twice/1 day)	TER, ADs	3 months	NR
Dang C, 2019 [42]	20	20	24.49 [6.62]				29.82 [13.35] months				NR	Yes	(A) (1) Fire needles (once/7 days); (2) plus (B)	(B) (1) Mometasone furoate (once/1 day; Shanghai schering-plough pharmaceutical Co., LTD); (2) NB-UVB (once/2 days); (3) compound betamethasone (1 ml/1 month, Schering-Plough Labo N.V. Belgium)	TER, TSLA, ADs	12 weeks	NR
He G, 2019 [43]	35	35	27 [8.2]	25 [9.3]			2.2 [1.5] years	2.5 [1.8] years			NR	Yes	(A) (1) Fire needles (once/7 days); (2) moxa-moxibustion (30 min/once, once/1 day, location: local skin lesion)	(B) 0.1% tacrolimus ointment (J20100015, Astellas (China) Co.)	TER, ADs	12 weeks	NR
Liu X, 2018 [44]	45	40	34.6 [5.2]	34.6 [5.2]			NR	NR			NR	NR	(A) (1) Fire needles (once/7 days); (2) plus (B)	(B) NB-UVB (once/1 day)	TER, the satisfaction of patients	3 months	NR
Chen S. Y, 2018 [45]	20	20	15.14 [1.2]	15.2 [1.3]			2.8 [1.1] years	2.7 [1.1] years			NR	Yes	(A) (1) Fire needles (once/7 days); (2) 308-nm excimer laser (once/7 days, Chongqing DMGD co., XECL-308C)	(B) 308-nm excimer laser (twice/7 days, Chongqing DMGD Co., XECL-308C)	TER, ET	3 months	NR
Chen X, 2018 [46]	35	36	37.686 [13.45]	32.611 [11.79]			52.09 [54.82] months	31.61 [45.60] months			Liver-kidney yin deficiency; Qi-stagnancy and blood stasis	Yes	(A) (1) Fire needles (once/7 days); (2) plus (B)	(B) (1) Chinese materia medica preparation; (2) 0.1% tacrolimus ointment (twice/1 day)	TER, different syndrome effectiveness rate, VNS analysis, ADs	12 weeks	NR
Cheng K, 2018 [47]	20	20	38.65 [13.36]	39.75 [12.15]			26.83 [8.11] months	26.23 [9.01] months			NR	Yes	(A) (1) Fire needles (once/3 days); (2) plus (B)	(B) 308-nm excimer laser (twice/7 days, Shenzhen GSD Co.)	TER, DPER, DDER, DCER, ADs	72 days	NR
Fu F, 2019 [48]	30	30	31.9 [10.6]	32.5 [9.75]			24.7 [12.73] months	27.65 [14.21] months			Blockage of the vessel	Yes	(A) (1) Fire needles (once/7 days); (2) plus (B)	(B) “Jia Wei Tao Hong Si Wu” decoction of TCM (0.5 dose/once, twice/1 day)	TER, ADs	12 weeks	NR
Gu T, 2018 [49]	52	52	36.8	38.2			1 months–7 years	6 months–8 years			NR	Yes	(A) (1) Fire needles (once/7 days); (2) plus (B)	(B) 308-nm excimer laser (PhotoMedex, American, AL10000, twice/7 days)	TER, ADs	12 weeks	NR
He X. L, 2018 [50]	29	28	40.17 [12.73]	39.57 [13.20]			45.59 [42.88] months	26.89 [27.12] months			NR	Yes	(A) (1) Fire needles (once/7–10 days); (2) “Jia Wei Tao Hong Si Wu” decoction of TCM (200 ml/once, twice/1 day)	(B) (1)308-nm excimer laser (twice/7 days) (2) “Jia Wei Tao Hong Si Wu” decoction of TCM (200 ml/once, twice/1 day)	TER, TSLA, DDER, QLQI score, ADs	90 days	NR
Li B, 2018 [51]	29	28	18.2 [2.3]	17.6 [2.1]			NR	NR			NR	Yes	(A) (1) Fire needles (once/7 days); (2) moxa-moxibustion (15 min/once, once/1 day, location: local skin lesion, Xiabai acupoint, Dianfeng acupoint, Zusanli acupoint)	(B) Halometasone cream (once/1 day; intermittent treatment: stopping for 1 week after using for 1 week.)	TER, TSLA, ADs	3 months	NR
Liu Y, 2019 [52]	31	31	34.2 [3.9]	33.9 [3.6]			2.5 [0.7]	2.4 [0.6]			Qi-stagnancy and blood stasis	Yes	(A) (1) Fire needles (once/7 days); (2) “Jia Wei Ru Yi Hei Bai” decoction of TCM (0.5 dose/once, twice/1 day)	(B) 0.03% tacrolimus ointment (twice/1 day, Astellas (China) Co.)	TER, TPP, CL, FUP, ADs	3 months	3 months
Reng S. S, 2018 [53]	24	24	34.69 [3.59]				3.29 [1.10]				Liver-kidney yin deficiency	Yes	(A) (1) Fire needles (7–10 days/1 month); (2) plus (B)	(B) (1) “Qu Yu Bu Shen” decoction of TCM (200–300 ml/once, twice/1 day); (2) tacrolimus ointment (twice/1 day)	TER, TRCR, ADs	6 months	NR
Xia F, 2019 [54]	24	23	37.0 [14.2]		37.3 [14.8]		4.6 [4.1]	4.3 [4.0]			NR	Yes	(A) (1) Fire needles (1^st^ month: once/3 days; 2^nd^ month: once/5 days; 3^rd^ month: once/7 days); (2) plus (B)	(B) 0.1% tacrolimus ointment (twice/1 day, Astellas (China) Co.)	TER, TSLA, FUP, TPP	3 months	6 months
Zhang F. R, 2018 [55]	34	33	32.65 [14.00]		31.42 [13.81]		32.50 [39.55]	36.03 [34.06]			NR	Cold and blood stasis resistance	(A) (1) Fire needles (once/7 days); (2) “Wen Jing” decoction of TCM (200 ml/once, twice/1 day) (3) plus (B)	(B) 0.1% tacrolimus ointment (twice/1 day, Astellas (China) Co.)	TER, TPP, Dermoscopy dynamic monitoring, TSLA, FUP, ADs	12 weeks	3 months
Zhao X. M, 2019 [56]	45	45	33.5 [3.8]		33.8 [3.5]		2.2 [0.7]	2.3 [0.8]			NR	Yes	(A) (1) Fire needles (once/7 days); (2) “Xiao Ban” tincture of TCM (3-4 times/1 day); (3) plus (B)	(B) Halometasone cream (once/1 day; intermittent treatment: stopping for 1 week after using for 2 weeks, Hongkong Bright Future Co., HC20100039)	TER, TPP, TSLA, CL, ADs	6 months	NR

SD, standard deviation; TCM, traditional Chinese medicine; E, experimental group; C, control group; ADs, adverse events; FUP, follow-up period; NR, not reported; TER, total effectiveness rate; DPER, different position effectiveness rate; DAER, different acreage effectiveness rate; DDER, different duration effectiveness rate; DCER, different classification effectiveness rate; ET, effectual time; TSLA, total skin lesion area; DPSLA, different position skin lesion area; s-CLSM, skin-confocal laser scanning microscope; TRCA, total restored color area; TPP, total pigment point; LA, loss analysis; CA, compliance analysis; CL, cytokine level, VSS, Vitiligo symptom score; VNS, validation of the Vitiligo Noticeability Scale; QL.

**Table 2:** Meta-analysis of the total effectiveness rate in 47 trials.

Trial (first author, year of publication)	Experimental		Control		Weight (%)	Risk ratio
	Events	Total	Events	Total		M-H, fixed, 95% CI
(A) Trials calculating the total effectiveness rate according to the NP formula						

(1) Fire needle therapy + 308-nm excimer laser versus 308-nm excimer laser alone						
Chen S. Y, 2018	11	20	5	20	0.70	2.20 [0.93, 5.18]
Dun G, 2016	46	50	34	50	4.50	1.35 [1.10, 1.66]
Gu T, 2018	41	52	26	52	3.40	1.58 [1.16, 2.14]
Jiang M. J, 2016	23	30	15	30	2.00	1.53 [1.02, 2.31]
Kuang W. B, 2013	76	96	44	90	6.00	1.62 [1.28, 2.05]
Ren L. S, 2016	42	50	33	50	4.40	1.27 [1.01, 1.61]
Xu C. Q, 2018	35	40	20	40	2.60	1.75 [1.26, 2.44]
Yu C. D, 2017	23	30	17	28	2.30	1.26 [0.88, 1.81]
Zhu Y. Y, 2016	36	43	28	40	3.80	1.20 [0.94, 1.52]
Subtotal (95% CI)		411		400	29.80	1.46 [1.32, 1.61]
Total events	333		222			
Heterogeneity: Chi^2^ = 8.17, *df* = 8 (*P*=0.42); *I*^2^ = 2%						
Test for overall effect: *Z* = 7.56 (*P* < 0.00001)						

(2) Fire needle therapy + other TCM methods versus other TCM methods alone						
Bo Z. F, 2017	12	23	3	23	0.40	4.00 [1.30, 12.33]
Fu F, 2019	18	30	8	30	1.10	2.25 [1.16, 4.36]
Lin M, 2019	31	48	15	47	2.00	2.02 [1.27, 3.23]
Liu Z. H, 2019	45	60	34	60	4.50	1.32 [1.02, 1.73]
Xu D. P, 2016	20	29	11	29	1.50	1.82 [1.07, 3.08]
Yang D. K, 2014	21	30	12	29	1.60	1.69 [1.03, 2.77]
Zhao X, 2018	35	49	27	49	3.60	1.30 [0.95, 1.76]
Zhao Y, 2015	27	39	15	39	2.00	1.80 [1.15, 2.82]
Subtotal (95% CI)		308		306	16.60	1.66 [1.43, 1.94]
Total events	209		125			
Heterogeneity: Chi^2^ = 9.39, *df* = 7 (*P*=0.23); *I*^2^ = 25%						
Test for overall effect: *Z* = 6.51 (*P* < 0.00001)						

(3) fire needle therapy + tacrolimus ointment versus tacrolimus ointment alone						
Xia F, 2019	20	24	13	23	1.80	1.47 [0.99, 2.20]
Yang D, 2017	20	31	13	31	1.70	1.54 [0.94, 2.51]
Yang M, 2017	6	18	3	19	0.40	2.11 [0.62, 7.20]
Subtotal (95% CI)		73		73	3.90	1.57 [1.15, 2.14]
Total events	46		29			
Heterogeneity: Chi^2^ = 0.32, *df* = 2 (*P*=0.85); *I*^2^ = 0%						
Test for overall effect: *Z* = 2.81 (*P*=0.005)						

(4) Fire needle therapy + other treatments versus other treatments alone						
Chen G. F, 2017	20	27	9	26	1.20	2.14 [1.21, 3.80]
Chen X, 2018	9	33	12	36	1.50	0.82 [0.40, 1.69]
Dang C, 2019	15	20	9	20	1.20	1.67 [0.96, 2.88]
Du Y. S, 2019	19	40	15	38	2.00	1.20 [0.72, 2.01]
Liu X, 2018	29	45	17	40	2.40	1.52 [1.00, 2.31]
Mai L. X, 2017	20	31	5	23	0.80	2.97 [1.31, 6.73]
Reng S. S, 2018	22	24	16	24	2.10	1.38 [1.01, 1.87]
Tian L. Y, 2018	22	40	13	40	1.70	1.69 [1.00, 2.87]
Wang W. L, 2017	29	39	16	39	2.10	1.81 [1.19, 2.76]
Wang W. L, 2018	45	50	32	50	4.20	1.41 [1.12, 1.77]
Yin Y. Q, 2018	18	32	10	31	1.30	1.74 [0.96, 3.16]
Zhang F. R, 2018	17	34	13	33	1.70	1.27 [0.74, 2.18]
Zhao X. M, 2019	29	45	20	45	2.60	1.45 [0.98, 2.15]
Zhou R. X, 2012	68	100	40	100	5.30	1.70 [1.29, 2.24]
Subtotal (95% CI)		560		545	30.30	1.56 [1.39, 1.74]
Total events	362		227			
Heterogeneity: Chi^2^ = 10.84, *df* = 13 (*P*=0.62); *I*^2^ = 0%						
Test for overall effect: *Z* = 7.54 (*P* < 0.00001)						

(5) Fire needle therapy + other TCM methods versus phototherapy + same TCM methods						
He X. L, 2018	26	29	16	28	2.20	1.57 [1.11, 2.21]
Jing L. H, 2015	23	31	13	29	1.80	1.66 [1.05, 2.61]
Li X. S, 2017	34	60	22	60	2.90	1.55 [1.04, 2.30]
Subtotal (95% CI)		120		117	6.80	1.58 [1.25, 2.00]
Total events	83		51			
Heterogeneity: Chi^2^ = 0.05, *df* = 2 (*P*=0.97); *I*^2^ = 0%						
Test for overall effect: *Z* = 3.85 (*P*=0.0001)						

(6) Fire needle therapy + other TCM methods versus other traditional treatments						
He G, 2018	22	28	16	28	2.10	1.38 [0.95, 2.00]
He G, 2019	19	35	10	35	1.30	1.90 [1.04, 3.48]
Li B, 2018	22	29	12	28	1.60	1.77 [1.10, 2.84]
Liu J. J, 2015	22	31	14	29	1.90	1.47 [0.95, 2.28]
Liu Y, 2019	20	29	13	23	1.90	1.22 [0.79, 1.88]
Wang X, 2016	24	32	13	33	1.70	1.90 [1.19, 3.04]
Zhang J. Y, 2016	24	32	15	33	2.00	1.65 [1.08, 2.52]
Subtotal (95% CI)		216		209	12.50	1.59 [1.34, 1.88]
Total events	153		93			
Heterogeneity: Chi^2^ = 3.25, *df* = 6 (*P*=0.78); *I*^2^ = 0%						
Test for overall effect: *Z* = 5.27 (*P* < 0.00001)						
Total (95% CI)		1688		1650	100.00	1.55 [1.46, 1.65]
Total events	1186		747			
Heterogeneity: Chi^2^ = 34.54, *df* = 43 (*P*=0.82); *I*^2^ = 0%						
Test for overall effect: *Z* = 14.34 (*P* < 0.00001)						
Test for subgroup differences: Chi^2^ = 2.30, *df* = 5 (*P*=0.81), *I*^2^ = 0%						

(B) Trials calculating the total effectiveness rate according to the NVSL formula						

(1) Fire needle therapy + other treatments versus other treatments alone						
Cheng K, 2018	19	38	11	34	6.60	1.55 [0.86, 2.76]
Luo G. P, 2016	161	235	118	247	65.30	1.43 [1.23, 1.68]
Xie H. L, 2017	64	115	51	122	28.10	1.33 [1.02, 1.74]
Subtotal (95% CI)		388		403	100.00	1.41 [1.24, 1.61]
Total events	244		180			
Heterogeneity: Chi^2^ = 0.32, *df* = 2 (*P*=0.85); *I*^2^ = 0%						
Test for overall effect: *Z* = 5.12 (*P* < 0.00001)						

CI, confidence intervals; M-H, Mantel–Haenszel.

## Data Availability

All data generated during this study are included in this article and in the Supplementary Information files.

## Abbreviations

TCM: Traditional Chinese medicine
RCTs: Randomized controlled trials
NP formula: Effectiveness rate = (the number of cured patients + the number of markedly effective patients)/the number of total patients × 100%
NVSL formula: Effectiveness rate = (the number of cured vitiligo skin lesions + the number of markedly effective vitiligo skin lesions)/ the number of total vitiligo skin lesions × 100%
RRs: Risk ratios
CIs: Confidence intervals
MDs: Mean differences
NB-UVB: Narrow-band ultraviolet B light
AMMC: Activate amelanotic melanocyte
IL: Interleukin
vs: Versus.

## Supplementary Materials

Supplementary Materials Supplementary File 1. National standards of the People's Republic of China- fire acupuncture. Supplementary File 2. Preferred Reporting Items for Systematic Reviews and Meta-analyses checklist. Supplementary File 3. Meta-analysis of total restoration of the area's color. Supplementary File 4. Meta-analysis of the total increased pigment point. Supplementary File 5. Meta-analysis of the reduced serum interleukin-17 level. Supplementary File 6. Meta-analysis of the effectual time. Supplementary File 7. Meta-analysis of the therapy's effectiveness in different lesion locations. Supplementary File 8. Meta-analysis of adverse effects. Supplementary File 9. Meta-analysis of recurrence rates.
