# Supplementary figures and images for: A New Therapy for Vitiligo Using Fire Needles: A Systematic Review of Evidence from 3618 Subjects
Source: Evid Based Complement Alternat Med. 2020 Aug 27;2020:8492097. doi: 10.1155/2020/8492097 (PMC7474359; doi:10.1155/2020/8492097)

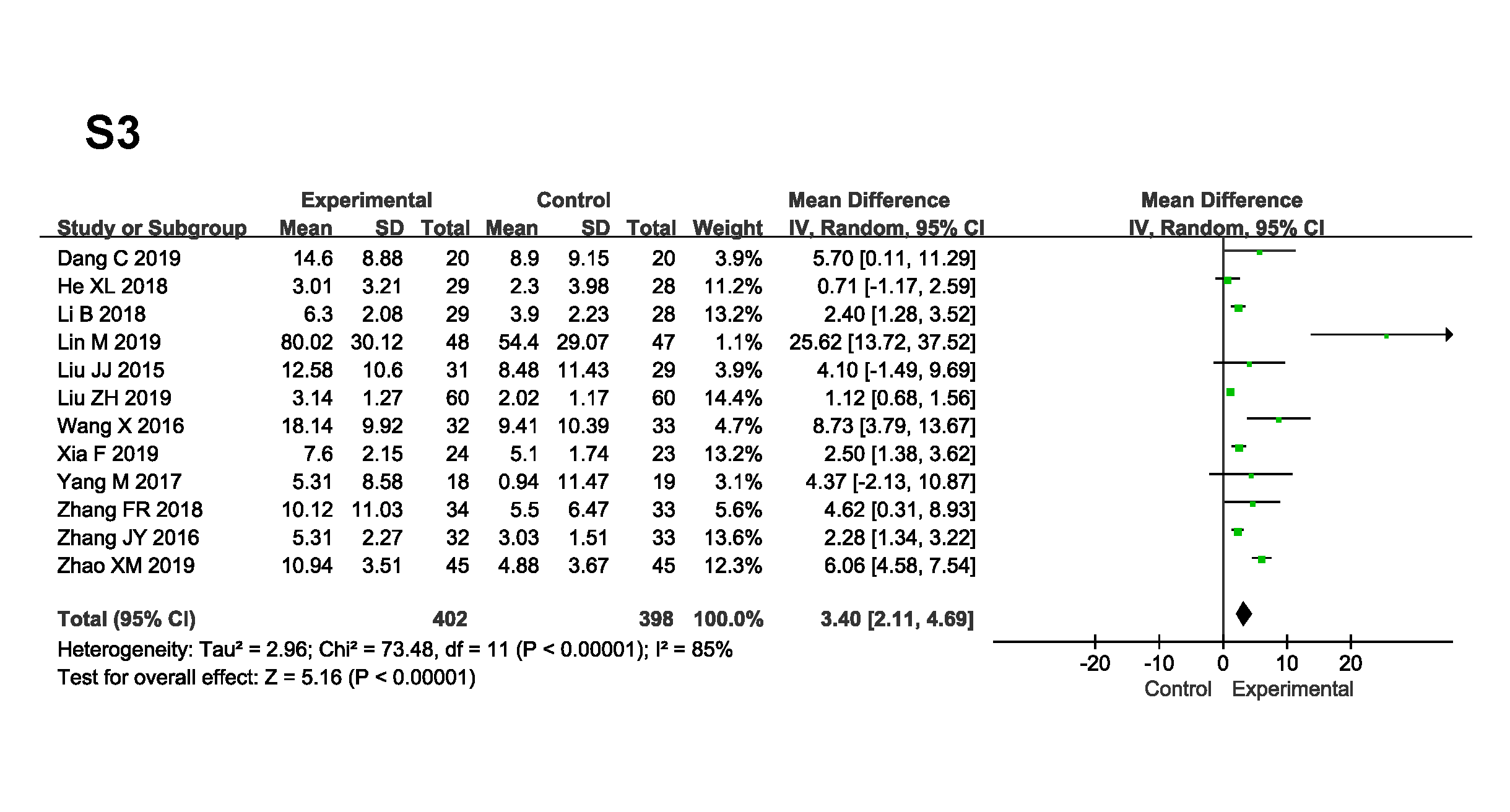

Supplement: Supplementary Materials — Supplementary File 1. National standards of the People's Republic of China- fire acupuncture. Supplementary File 2. Preferred Reporting Items for Systematic Reviews and Meta-analyses checklist. Supplementary File 3. Meta-analysis of total restoration of the area's color. Supplementary File 4. Meta-analysis of the total increased pigment point. Supplementary File 5. Meta-analysis of the reduced serum interleukin-17 level. Supplementary File 6. Meta-analysis of the effectual time. Supplementary File 7. Meta-analysis of the therapy's effectiveness in different lesion locations. Supplementary File 8. Meta-analysis of adverse effects. Supplementary File 9. Meta-analysis of recurrence rates. [file 8492097.f1.zip › 8492097.f1/Supplementary file 3.tiff]

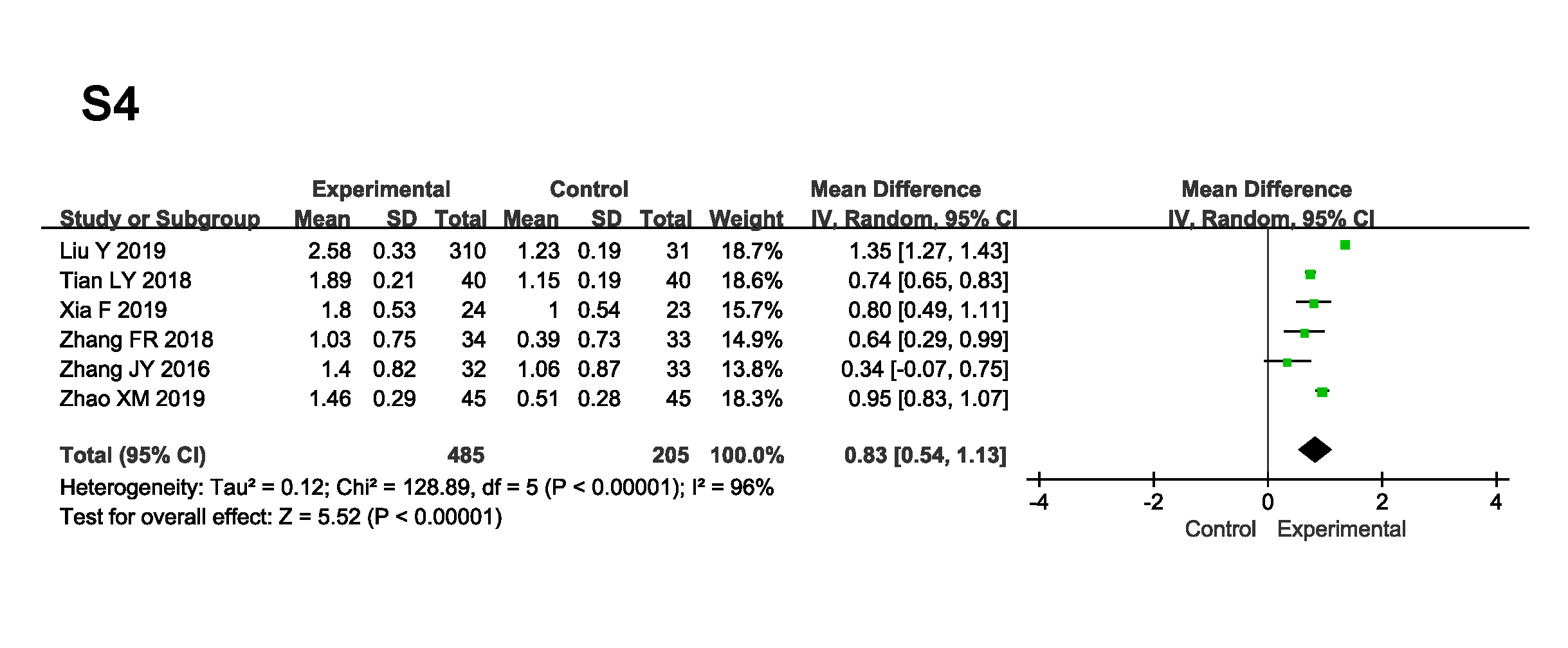

Supplement: Supplementary Materials — Supplementary File 1. National standards of the People's Republic of China- fire acupuncture. Supplementary File 2. Preferred Reporting Items for Systematic Reviews and Meta-analyses checklist. Supplementary File 3. Meta-analysis of total restoration of the area's color. Supplementary File 4. Meta-analysis of the total increased pigment point. Supplementary File 5. Meta-analysis of the reduced serum interleukin-17 level. Supplementary File 6. Meta-analysis of the effectual time. Supplementary File 7. Meta-analysis of the therapy's effectiveness in different lesion locations. Supplementary File 8. Meta-analysis of adverse effects. Supplementary File 9. Meta-analysis of recurrence rates. [file 8492097.f1.zip › 8492097.f1/Supplementary file 4.tiff]

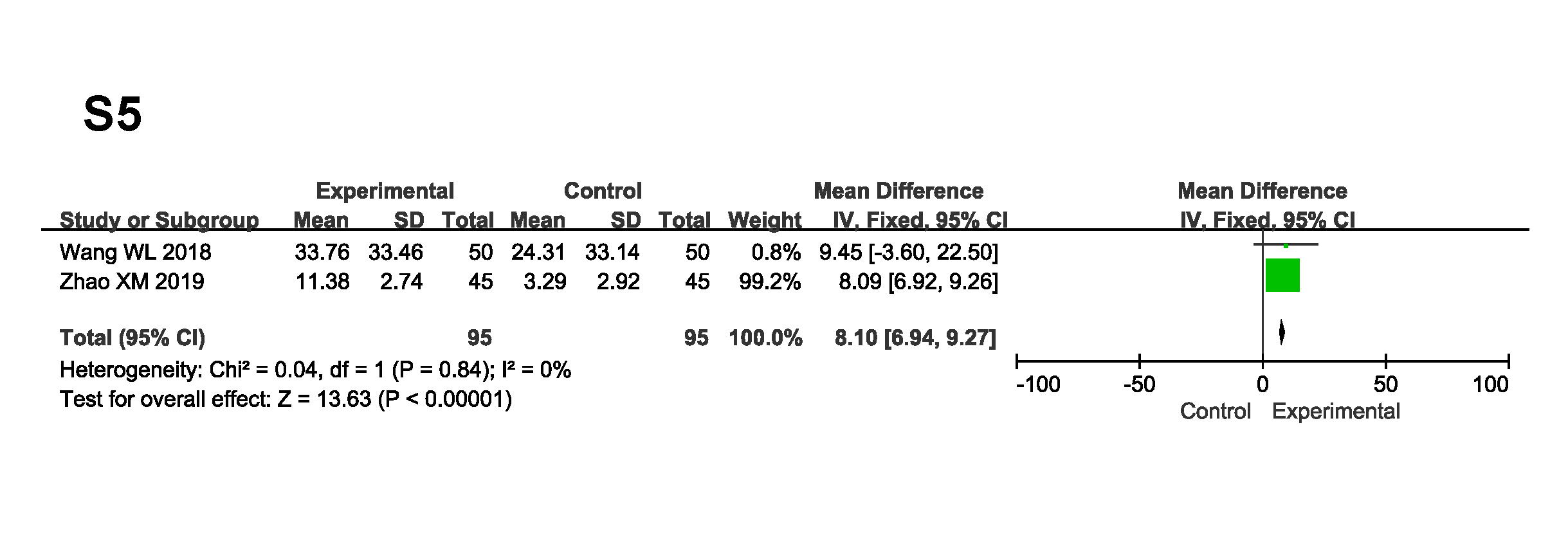

Supplement: Supplementary Materials — Supplementary File 1. National standards of the People's Republic of China- fire acupuncture. Supplementary File 2. Preferred Reporting Items for Systematic Reviews and Meta-analyses checklist. Supplementary File 3. Meta-analysis of total restoration of the area's color. Supplementary File 4. Meta-analysis of the total increased pigment point. Supplementary File 5. Meta-analysis of the reduced serum interleukin-17 level. Supplementary File 6. Meta-analysis of the effectual time. Supplementary File 7. Meta-analysis of the therapy's effectiveness in different lesion locations. Supplementary File 8. Meta-analysis of adverse effects. Supplementary File 9. Meta-analysis of recurrence rates. [file 8492097.f1.zip › 8492097.f1/Supplementary file 5.tiff]

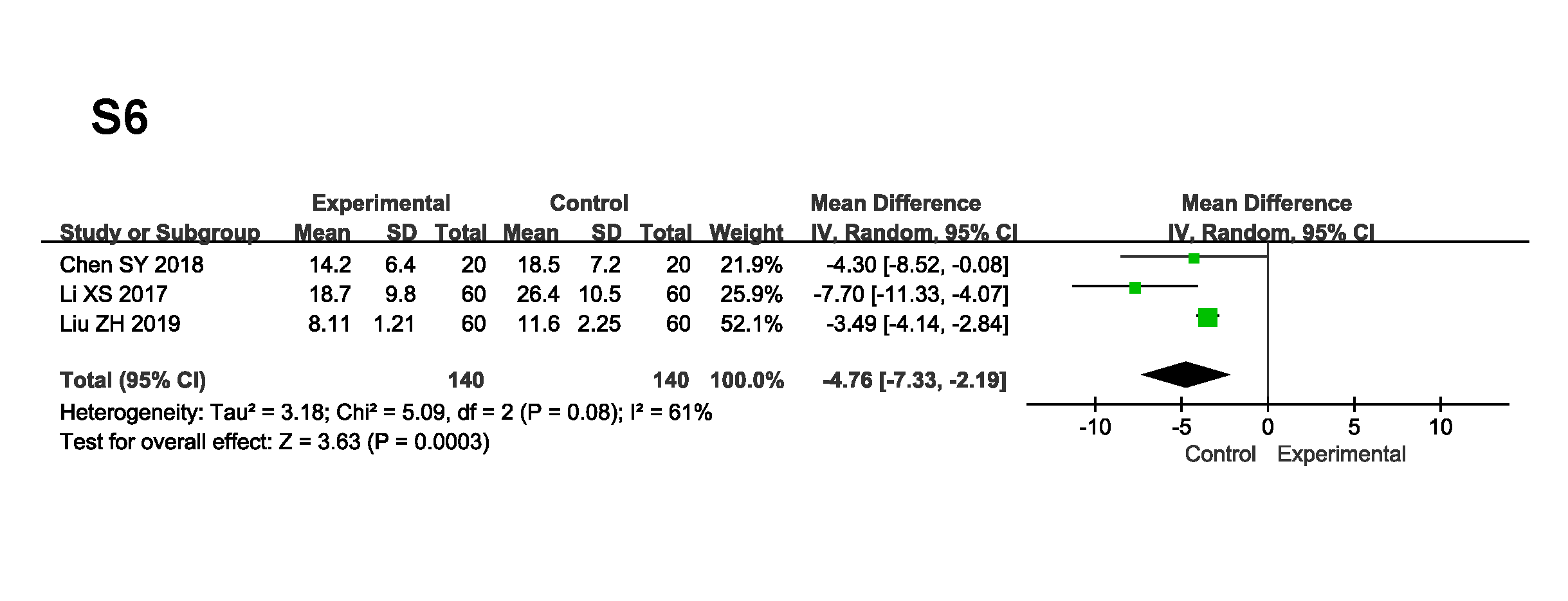

Supplement: Supplementary Materials — Supplementary File 1. National standards of the People's Republic of China- fire acupuncture. Supplementary File 2. Preferred Reporting Items for Systematic Reviews and Meta-analyses checklist. Supplementary File 3. Meta-analysis of total restoration of the area's color. Supplementary File 4. Meta-analysis of the total increased pigment point. Supplementary File 5. Meta-analysis of the reduced serum interleukin-17 level. Supplementary File 6. Meta-analysis of the effectual time. Supplementary File 7. Meta-analysis of the therapy's effectiveness in different lesion locations. Supplementary File 8. Meta-analysis of adverse effects. Supplementary File 9. Meta-analysis of recurrence rates. [file 8492097.f1.zip › 8492097.f1/Supplementary file 6.tiff]

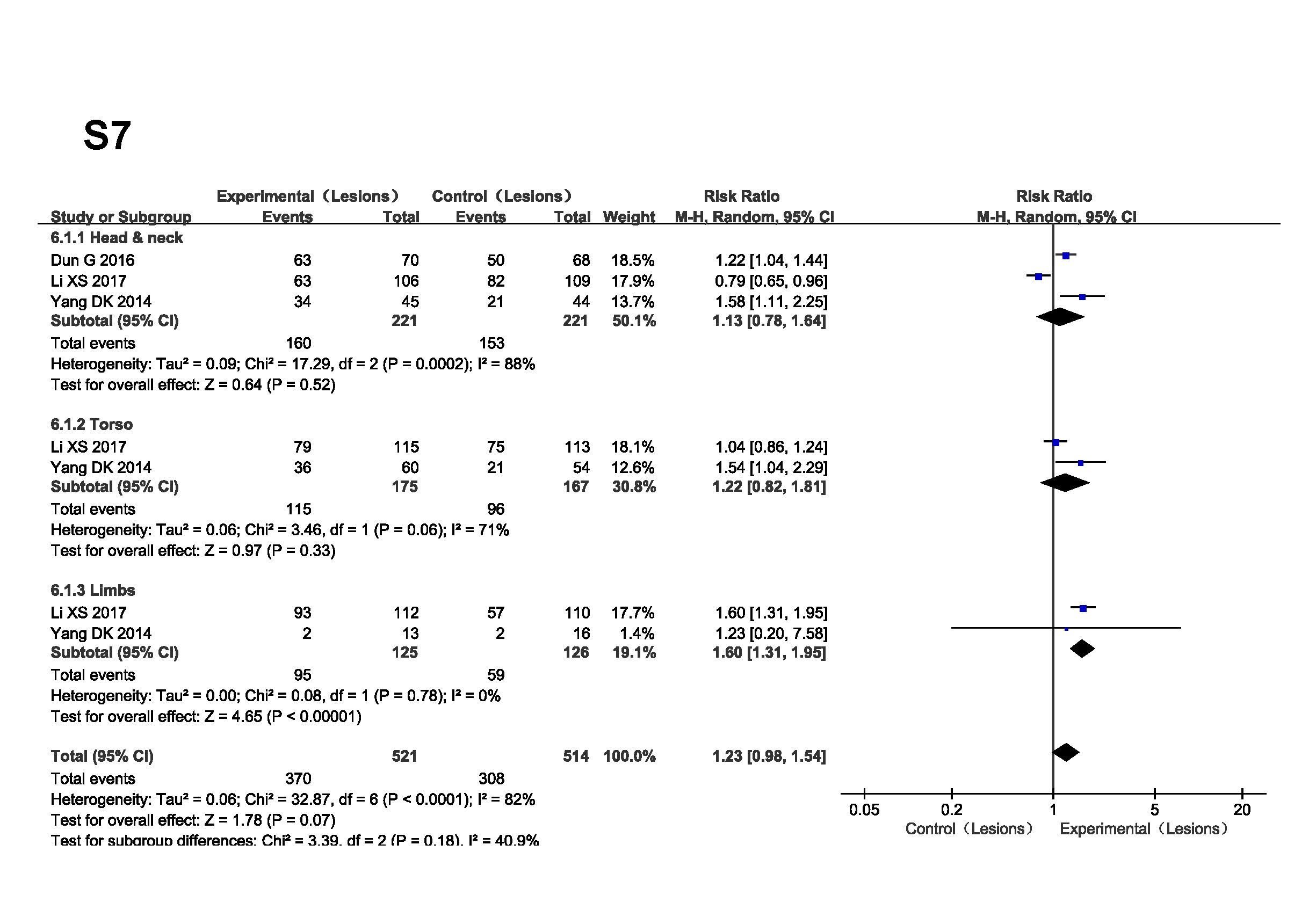

Supplement: Supplementary Materials — Supplementary File 1. National standards of the People's Republic of China- fire acupuncture. Supplementary File 2. Preferred Reporting Items for Systematic Reviews and Meta-analyses checklist. Supplementary File 3. Meta-analysis of total restoration of the area's color. Supplementary File 4. Meta-analysis of the total increased pigment point. Supplementary File 5. Meta-analysis of the reduced serum interleukin-17 level. Supplementary File 6. Meta-analysis of the effectual time. Supplementary File 7. Meta-analysis of the therapy's effectiveness in different lesion locations. Supplementary File 8. Meta-analysis of adverse effects. Supplementary File 9. Meta-analysis of recurrence rates. [file 8492097.f1.zip › 8492097.f1/Supplementary file 7.tiff]

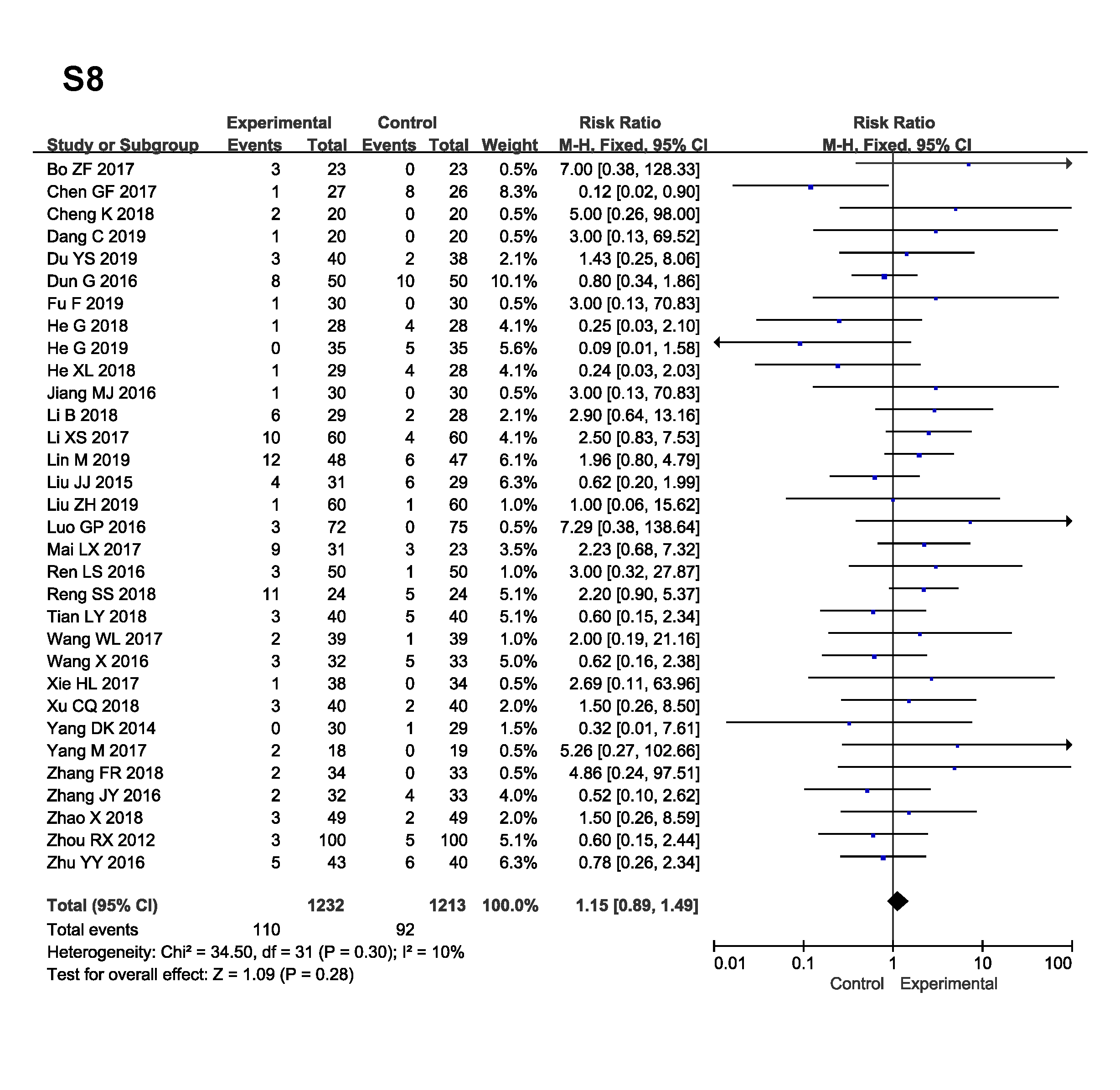

Supplement: Supplementary Materials — Supplementary File 1. National standards of the People's Republic of China- fire acupuncture. Supplementary File 2. Preferred Reporting Items for Systematic Reviews and Meta-analyses checklist. Supplementary File 3. Meta-analysis of total restoration of the area's color. Supplementary File 4. Meta-analysis of the total increased pigment point. Supplementary File 5. Meta-analysis of the reduced serum interleukin-17 level. Supplementary File 6. Meta-analysis of the effectual time. Supplementary File 7. Meta-analysis of the therapy's effectiveness in different lesion locations. Supplementary File 8. Meta-analysis of adverse effects. Supplementary File 9. Meta-analysis of recurrence rates. [file 8492097.f1.zip › 8492097.f1/Supplementary file 8.tiff]

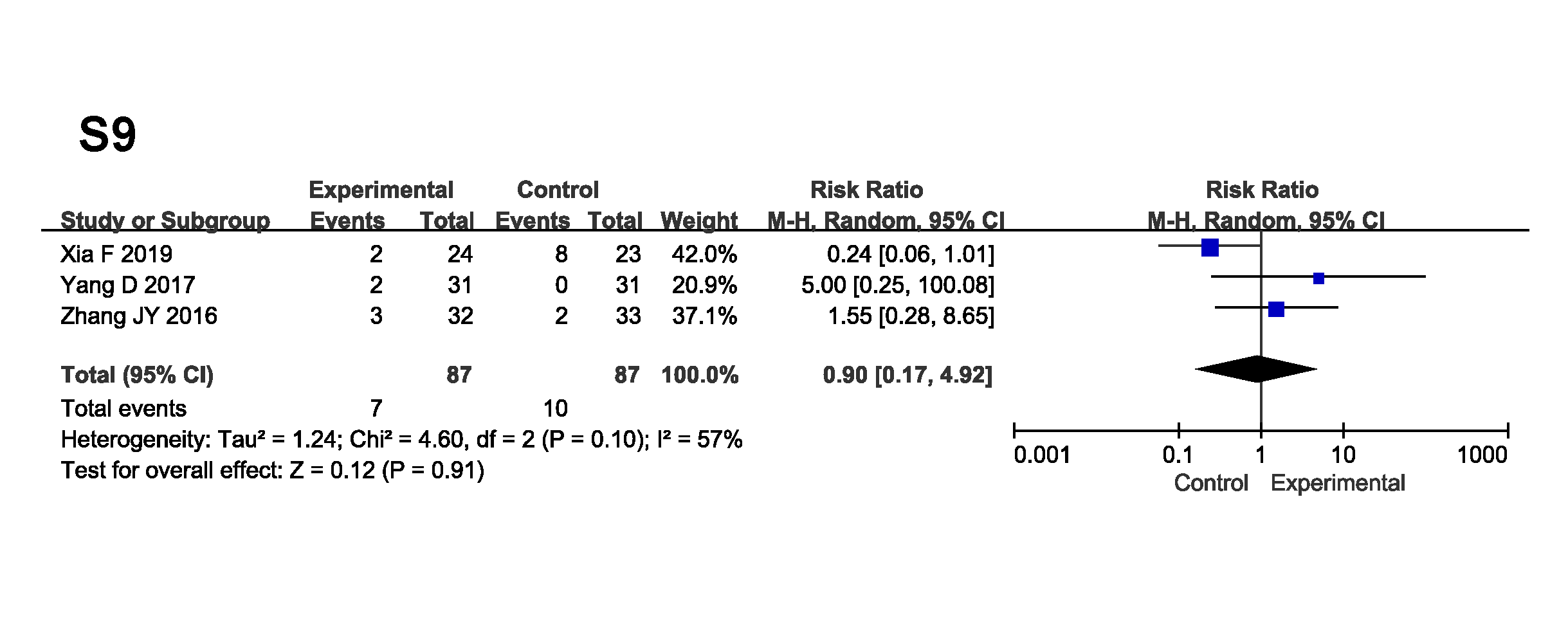

Supplement: Supplementary Materials — Supplementary File 1. National standards of the People's Republic of China- fire acupuncture. Supplementary File 2. Preferred Reporting Items for Systematic Reviews and Meta-analyses checklist. Supplementary File 3. Meta-analysis of total restoration of the area's color. Supplementary File 4. Meta-analysis of the total increased pigment point. Supplementary File 5. Meta-analysis of the reduced serum interleukin-17 level. Supplementary File 6. Meta-analysis of the effectual time. Supplementary File 7. Meta-analysis of the therapy's effectiveness in different lesion locations. Supplementary File 8. Meta-analysis of adverse effects. Supplementary File 9. Meta-analysis of recurrence rates. [file 8492097.f1.zip › 8492097.f1/Supplementary file 9.tiff]
