# Supplementary material for: A New Therapy for Vitiligo Using Fire Needles: A Systematic Review of Evidence from 3618 Subjects
Source: Evid Based Complement Alternat Med. 2020 Aug 27;2020:8492097. doi: 10.1155/2020/8492097 (PMC7474359; doi:10.1155/2020/8492097)
Supplement: Supplementary Materials — Supplementary File 1. National standards of the People's Republic of China- fire acupuncture. Supplementary File 2. Preferred Reporting Items for Systematic Reviews and Meta-analyses checklist. Supplementary File 3. Meta-analysis of total restoration of the area's color. Supplementary File 4. Meta-analysis of the total increased pigment point. Supplementary File 5. Meta-analysis of the reduced serum interleukin-17 level. Supplementary File 6. Meta-analysis of the effectual time. Supplementary File 7. Meta-analysis of the therapy's effectiveness in different lesion locations. Supplementary File 8. Meta-analysis of adverse effects. Supplementary File 9. Meta-analysis of recurrence rates. [file 8492097.f1.zip › 8492097.f1/Supplementary file 1.doc]

**National standards of the People's Republic of China**

Standardized manipulations of acupuncture and moxibustion - Part 12: Fire acupuncture

**1. Range**

This part of the GB/T 21709 specifies the terms and definitions, operating procedures and requirements, precautions, and contraindications for fire needle punching and fire needle operation.

This section applies to the technical operation of the fire acupuncture method.

**2. Terminology and definitions**

2.1 Fire needle: A metal that is resistant to high temperatures and harmless to the human body used as a needle for burning red.

2.2 Fire needle therapy: Acupuncture method of burning a red fire needle and quickly piercing a selected part of the human body according to a certain stab method.

**3. Operation steps and requirements**

**3.1 Preoperative preparation**

**3.1.1 Selection of instrument**

**3.1.1.1 Fire needle**

The tip of the needle should be round and free of barbs, the needle should be smooth and free of rust, and the needle handle should be securely connected to the needle. See Appendix A for the structure, material, and specifications of the fire needle.

**3.1.1.2 Heating tools**

Ignite the alcohol lamp for needle heating or heat the needle in other safe ways.

**3.1.2 Acupuncture points**

According to the patient's condition, choose acupuncture points on the face, meridians, body surface lesions, or around the lesions. Mark the selected acupuncture points to ensure the accuracy of the needling. See Appendix B for suggestions for fire acupuncture.

**3.1.3 Treatment posture**

According to the condition and the location of the acupuncture, the patient can choose a comfortable and safe position that allows the treatment to be performed easily.

**3.1.4 Environmental standards**

The treatment environment should be clean and devoid of flammable materials, but should not have a strong air flow.

**3.1.5 Disinfection**

**3.1.5.1 For the medical practitioner**

The hands of the doctor should be cleaned with soapy water and then wiped with 75% ethanol.

**3.1.5.2 For acupuncture points**

Use 75% ethanol or 0.5% to 1% crouched cotton balls to disinfect at the acupuncture points.

**3.1.5.3 For fire needles**

Ignite the alcohol lamp, continuously move the red from the needle root to the needle tip and disinfect the needle before the operation.

**3.2 Methods of operation**

**3.2.1 Heating the needle**

Burn the red needle tip and the needle body with an alcohol lamp and determine the length of the red needle to burn according to the depth of the needle.

**3.2.2 Puncturing**

After the needle is burnt red, it should be quickly and accurately punctured into the acupuncture points.

**3.2.3 Common methods of acupuncture**

3.2.3.1 A single needle puncture is applied to the acupuncture points on the face.

3.2.3.2 Multi-needle intensive stimulation is applied to the surface lesions at intervals of no more than 1 cm per needle.

3.2.3.3 Multi-needle evacuation stimulation is applied to the surface lesions at intervals of about 2 cm per needle.

3.2.3.4 Acupuncture points are placed at the junction of the lesion and normal tissue around the surface of the lesion.

3.2.3.5 A fire needle is used to puncture a stagnant blood vessel at the body surface to release an appropriate amount of blood.

**3.2.4 Taking out the needle**

After the needle reaches the treatment depth, the needle can be released.

**3.3 Postoperative treatment**

**3.3.1 Disinfection**

To avoid cross-infection caused by the needle body, the alcohol lamp should be used to continuously moved from the needle root along the needle body to the needle tip; the needle should also be disinfected after use.

**3.2 Pinhole care**

To relieve pain and promote healing, pinholes should be treated in the following manner:

(a) Press the pinhole with a sterile cotton ball or cotton swab;

(b) If there is bleeding or exudate in the pinhole, wipe it with a sterile cotton ball;

(c) When fire needle treatment causes bleeding in a blocked venous blood vessel, wait until the bleeding stops before wiping with a sterile cotton ball.

**4. Notices**

4.1 Pay attention to potential hazards during operation to prevent accidents such as burns or fires.

4.2 Acupuncture should avoid arteries and nerve trunks; internal organs and vital organs should not be damaged.

4.3 Pregnant women, puerperal individuals, and infants should be treated with caution.

4.4 Patients with diabetes, scars, or allergies should be treated with caution.

4.5 Patients who are too nervous, hungry, and tired should not use fire needle therapy.

4.6 After the operation, the doctor should explain to the patient the maintenance of the post-acupuncture site, including:

a) If the pinhole is partially red, hot, mildly painful, or itching, etc., it is normal and may not last long;

b) During local cleaning, the pinhole should not be scratched by hand; avoid getting oil or cream near the pinhole;

c) Pinholes should not be exposed to water on the same day.

**5 Contraindications**

5.1 Unexplained masses cannot be treated with fire needles.

5.2 Patients with major blood loss and coagulation disorders should not be treated with fire needles.
